# Characterization of a Novel Cutaneous Human Papillomavirus Genotype HPV-125

**DOI:** 10.1371/journal.pone.0022414

**Published:** 2011-07-21

**Authors:** Anja Kovanda, Boštjan J. Kocjan, Marko Potočnik, Mario Poljak

**Affiliations:** 1 Institute of Microbiology and Immunology, Faculty of Medicine, University of Ljubljana, Ljubljana, Slovenia; 2 Department of Dermatovenereology, University Medical Centre Ljubljana, Ljubljana, Slovenia; National Cancer Institute, United States of America

## Abstract

The DNA genome of a novel HPV genotype, HPV-125, isolated from a hand wart of an immuno-competent 19-year old male was fully cloned, sequenced and characterized. The full genome of HPV-125 is 7,809-bp in length with a GC content of 46.4%. By comparing the nucleotide sequence of the complete L1 gene, HPV-125 is phylogenetically placed within cutaneotrophic species 2 of *Alphapapillomaviruses*, and is most closely related to HPV-3 and HPV-28. HPV-125 has a typical genomic organization of *Alphapapillomaviruses* and contains genes coding for five early proteins, E6, E7, E1, E2 and E4 and two late capsid proteins, L1 and L2. The genome contains two non-coding regions: the first located between the L1 and E6 genes (nucleotide positions 7,137–7,809, length 673-bp) and the second between genes E2 and L2 (nucleotide positions 3,757–4,216, length 460-bp). The E6 protein of HPV-125 contains two regular zinc-binding domains at amino acid positions 29 and 102, whereas the E7 protein exhibits one such domain at position 50. HPV-125 lacks the regular pRb-binding core sequence within its E7 protein. In order to assess the tissue predilection and clinical significance of HPV-125, a quantitative type-specific real-time PCR was developed. The 95% limit-of-detection of the assay was 2.5 copies per reaction (range 1.7–5.7) and the intra- and inter-assay coefficients of variation were 0.47 and 2.00 for 100 copies per reaction, and 1.15 and 2.15 for 10 copies per reaction, respectively. Testing of a representative collection of HPV-associated mucosal and cutaneous benign and malignant neoplasms and hair follicles (a total of 601 samples) showed that HPV-125 is a relatively rare HPV genotype, with cutaneous tropism etiologically linked with sporadic cases of common warts.

## Introduction

Papillomaviruses (PV) are small non-enveloped viruses with a double stranded circular DNA genome, approximately 8-kb in size. So far, 29 genera of papillomaviruses, designated by letters of the Greek alphabet, have been described, of which 5 genera (*Alpha*-, *Beta*-, *Gamma*-, *Mu*- and *Nupapillomavirus*) contain members that can infect humans and are referred to as human papillomaviruses (HPV) [1], [2]. HPV genotypes that represent natural papillomavirus taxonomic entities, are grouped into HPV species according to their tissue tropism, sequence similarity, and other distinct biological properties. In order to be recognized as a distinct genotype, the entire L1 open reading frame (ORF) of a candidate HPV isolate must differ by at least 10% from all other officially recognized HPV genotypes [1], [2] and the entire genome of the novel genotype must be deposited, in the form of one or more clones, in the Reference Centre for Papillomaviruses in Heidelberg, Germany, so that the full genomic sequence can be independently confirmed. If these two conditions are met, the novel genotype is assigned a consecutive number, based on its order of submission, by the Reference Centre.

To date, more than 150 HPV genotypes have been fully characterized; roughly a third of them have been discovered since 2004, mostly by the use of molecular methods [1], [2]. Based on the large number of partial sequences published in the last few years, the final characterization of many further new HPV genotypes is expected in the near future [3], [4]. The majority of officially recognized HPV genotypes have been etiologically linked to specific diseases, such as various forms of cancer, common and genital warts and other benign and malignant mucosal and skin lesions [5]. Most notably, infection with several genotypes of *Alphapapillomavirus* species 7 (type species HPV-18) and 9 (type species HPV-16) is strongly associated with the development of cervical carcinoma and other cancers in the anogenital region of both genders [5], [6], while infection with members of *Alphapapillomavirus* species 10 (type species HPV-6) is associated with the development of benign tumors such as genital warts and laryngeal papillomas [5]. Another common HPV-associated clinical entity, *verrucae vulgaris*, or common warts, is caused by members of *Alphapapillomavirus* species 2 and 4, and several species of *Gama*-, *Mu*- and *Nupapillomavirus* genera [7]. Members of *Alphapapillomavirus* species 2 were first found in patients with the hereditary disorder *epidermodysplasia verruciformis* [8] and are most frequently associated with flat or plane and intermediate skin warts in immuno-competent individuals [9]–[11]. In immuno-suppressed patients, such as solid-organ recipients, these genotypes are also associated or can co-localize with dysplastic warts and non-melanoma skin cancer [12]–[15].

In this study, a novel HPV genotype, isolated originally from a hand wart (isolate SIBX9) and initially characterized by our group in 2004 [16], was characterized fully and deposited in the Reference Centre for Papillomaviruses in Heidelberg, Germany, where it was assigned its official name, HPV-125. In addition, a quantitative type-specific real-time PCR (RT-PCR) was developed and a representative collection of HPV-associated benign and malignant neoplasms and hair follicles was tested in order to assess the tissue predilection and clinical significance of HPV-125.

## Materials and Methods

### Amplification and sequencing of initial 474-bp sequence of the HPV-125 L1 gene

The total DNA from the original clinical sample of a hand wart containing HPV-125 was extracted using a High Pure PCR Template Preparation kit (Roche Applied Science, Mannheim, Germany), according to the manufacturer's instructions [16]. The initial 474-bp sequence of the HPV-125 L1 gene (GenBank Acc. No: AJ810860, corresponding to nucleotide positions 5,994–6,468 of the HPV-125 complete genome) was obtained by the use of primers HVP2 (5′-TCNMGNGGNCANCCNYTNGG-3′ [14]) and B5 (5′- AYNCCRTTRTTRTGNCCYTG -3′ [13]) and FastStart Taq DNA polymerase kit (Roche Applied Science) on a PE9700 Thermo Cycler (Applied Biosystems, Foster City, CA). PCR was carried out in a 25 µl reaction volume containing 5 µl (100 ng) of extracted DNA, 2.5 µl of 10× PCR Reaction Buffer, 200 µM (each) of dATP, dCTP, dGTP, and dTTP, 1.5 mM of MgCl_2_, 1.25 U of FastStart Taq DNA Polymerase, and 25 pmol of each primer. The thermal cycler program was set to 4 min at 94°C, followed by 40 cycles consisting of 1 min at 95°C, 2 min at 52°C and 1 min at 72°C. The final extension step was performed at 72°C for 4 min and the reaction mixtures were then cooled to 4°C. Sequencing of the 474-bp PCR fragment was done by using the primers HVP2 and B5 on the ABI Prism® 310 Genetic Analyzer System (Applied Biosystems) and Big Dye® Terminator v 1.1 Cycle Sequencing Kit (Applied Biosystems).

### Amplification, sequencing and cloning of the complete genome of HPV-125

Primers for the reverse long template PCR (125-fpw2 and 125-rpw2, Table S1) were constructed manually on the basis of the previously obtained 474-bp sequence of the HPV-125 L1 gene. A 7,770-bp PCR fragment was obtained from the original clinical sample by using the Expand Long Template PCR System (Roche Applied Science) on a PE9700 Thermo Cycler (Applied Biosystems). PCR was carried out in a 25 µl reaction volume containing 7.1 µl of extracted DNA, 2.5 µl of 10× Expand Long Template buffer 2 with 27.5 mM MgCl_2_, 500 µM (each) of dATP, dCTP, dGTP, and dTTP, 0.75 U of Expand Long Template Enzyme Mix, and 3 pmol of each primer. The thermal cycler program was set to 2 min at 94°C, followed by 40 cycles consisting of 10 s at 94°C, 30 s at 58°C and 9 min at 68°C. The final extension step was performed at 68°C for 10 min and the reaction mixtures were then cooled to 4°C. Sequencing of the 7,770-bp PCR fragment was done by primer walking with 38 primers (Table S1) by using the ABI Prism® 310 Genetic Analyzer System (Applied Biosystems) and Big Dye® Terminator v 1.1 Cycle Sequencing Kit (Applied Biosystems). The full genome sequence of HPV-125 was deposited in GenBank through EMBL in September 2009 and can be accessed under acc. no. FN547152.

PCR fragments for the cloning of the full genome of HPV-125 were prepared with the Expand Long Template PCR System (Roche Applied Science) from the original clinical sample using sequencing primer pairs 125-fpw5 and 125-rpw6, and 125-fpw14 and 125-rpw 15 (Table S1), giving 4,594-bp and 5,140-bp overlapping PCR fragments, respectively. Both PCR fragments were sequenced and their sequences were found to be identical to that of the larger 7,700-bp PCR fragment. Both PCRs were carried out in a 25 µl reaction volume containing 5 µl of extracted DNA, 2.5 µl of 10× Expand Long Template buffer 2 with 27.5 mM MgCl_2_, 500 µM (each) of dATP, dCTP, dGTP, and dTTP, 0.75 U of Expand Long Template Enzyme Mix, and 3 pmol of each primer. The thermal cycler program was set to 2 min at 94°C, followed by 40 cycles consisting of 10 s at 94°C, 30 s at 56°C and 6 min at 68°C. The final extension step was performed at 68°C for 10 min and the reaction mixtures were then cooled to 4°C.

Before cloning, PCR products were run on gel electrophoresis, excised and purified from 1% agarose gel using a GeneJET™ Gel Extraction Kit (Fermentas, Vilnius, Lithuania). Plasmid clones containing overlapping PCR products were prepared using a CloneJET™ PCR Cloning Kit (Fermentas) according to the manufacturer's instructions. TransformAid™ Bacterial Transformation Kit (Fermentas) was used to transform *E.coli* strain JM107 with the plasmids, according to the manufacturer's instructions.

Plasmid clones isolated from the transformed overnight bacterial culture with the GeneJET™ Plasmid Miniprep Kit (Fermentas) were checked for correct inserts by size analysis and sequencing with the pJET1.2 sequencing primers, according to the manufacturer's instructions. Full sequencing by primer walking was carried out for two clones, confirming the previously determined full sequence of the HPV-125 genome. Both clones, together covering the full genome of HPV-125, were deposited in the Reference Centre for Papillomaviruses, Heidelberg, Germany in September 2009, where they were re-sequenced and the novel genotype was assigned its number in October 2009.

### ORF and phylogenetic analysis

The ORFs of HPV-125 were determined using the ORF finder function of Vector NTI Advance™ v 11.0 (Invitrogen, Carlsbad, CA) and pairwise comparison of genes of closely related HPV genotypes.

In order to phylogenetically place HPV-125, a multiple sequence alignment of L1 ORF of HPV-125 and 116 previously characterized HPV types available [1], [2], was constructed using the Alignment explorer program of the MEGA4 software package [17], [18], at the nucleotide level. All positions containing gaps and missing data were eliminated from the alignment, leaving 1,171 nucleotide positions in the final dataset.

All further phylogenetic analyses were also conducted by using MEGA4 [17], [18]. The evolutionary history was inferred by using the Minimum Evolution (ME) method [19]. The evolutionary distances (in units of the number of base substitutions per site) were computed by using the Maximum Composite Likelihood method [18] and the Neighbor-joining algorithm [20] was used to generate the initial tree. The ME tree was searched using the Close-Neighbor-Interchange algorithm [21] at a search level of 1.

**Figure 1:**
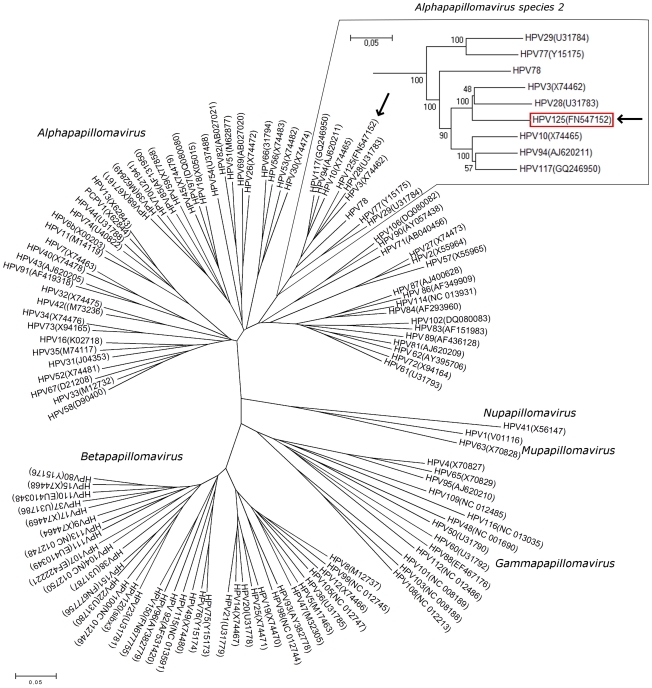
A consensus phylogenetic tree of 117 HPV L1 sequences. The evolutionary history was inferred using the Minimum Evolution method [19] and the consensus tree was inferred from 1,000 bootstrap replicates. The evolutionary distances were computed using the Maximum Composite Likelihood method [18] and are in units of the number of base substitutions per site. Bootstrap values for members of *Alphapapillomavirus* species 2 are given above the main tree. The GenBank account numbers of the HPV genotypes are given next to the genotypes' name, where available.

The optimal tree with the sum of branch length = 14,14565449 was used for Figure 1, the tree remaining drawn to scale, with branch lengths in the same units as those of the evolutionary distances used to infer the phylogenetic tree. The tree was bootstrap re-sampled 1,000 times.

Additionally, Maximum Parsimony analysis of the same dataset was conducted (data not shown), using the Close-Neighbor-Interchange algorithm to search for the optimal tree, also by using the MEGA 4 software package, which also grouped HPV-125 within the *Alphapapillomavirus* species 2.

In order to compare HPV-125 more closely with other genotypes of the *Alphapapillomavirus* species 2, pairwise sequence alignments (both nucleotide and amino acid) were carried out and percentage similarity was calculated for each of the viral genes (E6, E7, E1, E2, L1 and L2) between HPV-125 and each of the genotypes for which full genomic sequences were available (HPV-3, HPV-10, HPV-28, HPV-29, HPV-77, HPV-94 and HPV-117), by using the BioEdit v7.0.9.0. software package [22].

### HPV-125 type specific real-time PCR

The primers 125-RT-L1-F (5′- GGTTACCCGACCCAAATAAGT -3′, nucleotide positions 5,851–5,871) and 125-RT-L1-R (5′- TCGGCGTCAGGATTATAGATG -3′, nucleotide positions 5,891–5,911), specific for HPV-125, were chosen within the L1 gene by using the on-line ProbeFinder software (Roche Applied Science). FAM-labeled Universal Probe nr. 14 (Roche Applied Science) was used to detect the specific products in the real-time reaction.

The plasmid reference clone of HPV-125, containing the entire L1 sequence, was used to optimize the RT-PCR conditions and to evaluate the linearity, lower detection limit and reproducibility of the assay. The number of starting plasmid copies/µl was calculated from the concentration of plasmid DNA, quantified using a NanoDrop ND-1000 spectrophotometer (Nanodrop Technologies, Oxfordshire, UK), as described previously [23].

The optimized RT-PCR reaction mixture contained 10 µl of LightCycler® Probes Master 2× conc. (Roche Applied Science), 0.2 µM of each primer, 0.05 µM of Universal Probe nr. 14 (Roche Applied Science), 1 U of LightCycler® Uracil-DNA Glycosylase (Roche Applied Science), sample DNA and water up to a final volume of 20 µl. The assay was performed on a LightCycler® 480 real-time PCR Instrument (Roche Applied Science) under the following conditions: Uracil-N-Glycosylase activation at 40°C and pre-incubation at 95°C for 10 min each, followed by 45 cycles at 95°C for 10 s (denaturation) and 60°C for 30 s (annealing/extension). Acquisition of the fluorescence signal (FAM 465–510 nm) was performed during each annealing/extension step. The final cooling step consisted of a 10 s hold at 40°C.

To evaluate the linearity of the RT-PCR assay, ten-fold serial dilutions of the reference plasmid were prepared in 1.5 ml DNA LoBind tubes (Eppendorf, Hamburg, Germany) using a water solution with carrier RNA (1 µg/ml) and tested in triplicate. The limit of detection of the assay was determined by testing 15 replicates of the reference plasmid dilutions corresponding to an input of 25, 13, 6, 3, 1.5, 0.8 and 0.4 copies per reaction and calculated by Probit analysis using SPSS software v.15.0.0.0 (SPSS GmbH Software, Munich, Germany). Intra- and inter-assay reproducibility of the RT-PCR were assessed by testing triplicates of plasmid dilutions corresponding to an input of 100 and 10 copies per reaction, in three independent runs.

To ensure specificity and to check for cross-reactivity of RT-PCR, 10^7^ to 10^8^ copies of reference clones or synthetic L1 sequences (inserted into plasmid pUC57, by Genscript, Piscataway, USA) of HPV-2, HPV-3, HPV-7, HPV-10, HPV-27, HPV-28, HPV-29, HPV-40, HPV-43, HPV-77, HPV-78, HPV-91 (kindly provided by E-M. de Villiers, T. Matsukura and R. Burk) were tested.

### Clinical samples

The tissue predilection and clinical significance of HPV-125 were assessed on a representative collection of HPV-associated benign and malignant neoplasms and hair follicles. The DNA was isolated from a total of 601 samples obtained from the same number of patients, including tissue samples of histologically confirmed cervical squamous cell carcinoma (104), laryngeal papilloma (85), genital warts (71), common warts (102), squamous cell carcinoma (SCC) of the skin (52) basal cell carcinoma (BCC) of the skin (49) and hair follicles (138), as described previously [24], [25]. The quality of isolated DNA and absence of amplification inhibitors were determined by testing for the 268-bp or 110-bp fragment of human beta-globin using RT-PCR, as described previously [24]. The number of diploid human cells for the HPV-125 positive tissue samples was calculated from the beta-globin concentration, as described previously [26]. All positive results of the HPV-125 RT- PCR assay were confirmed by sequencing.

The presence of additional HPV genotypes in HPV-125 positive samples was determined using two commercial line-probe assays: RHA skin (beta) HPV (Diassay B.V., Rijswijk, The Netherlands), which allows identification of 26 different *Betapapillomavirus* HPV genotypes, and INNO-LiPA HPV Genotyping Extra (Innogenetics, Gent, Belgium), which allows identification of 28 different *Alphapapillomavirus* HPV genotypes, and additionally by three consensus in-house PCRs using Ma/Ha [27], CPI/CPIIs [28] and GP 5+/6+ [29] primers and sequencing of PCR-products.

### Ethics Statement

None of the 601 clinical samples were collected solely for the purpose of this study. All samples included in the study were collected in compliance with the Helsinki declaration. Samples of cervical cancer, hair follicles, genital warts and common warts were collected prospectively in our previous studies. These studies were approved by the Ethics Committee of the Ministry of Health of Republic of Slovenia (Consent references 34/11/06, 83/11/09, 174/05/09, 97/11/09 and 100/12/09) and written informed consent was obtained for each patient. Samples of laryngeal papillomas, squamous cell carcinoma and basal cell carcinoma of the skin were retrieved from the tissue collection of paraffin-embedded samples of the Institute of Pathology, Faculty of Medicine, University of Ljubljana. Approval from Institutional Review Board of the Institute of Pathology, Faculty of Medicine, University of Ljubljana was obtained prior to starting work on samples included in this study. In accordance with national legislation of the Republic of Slovenia no informed consent is needed for research on archival clinical samples. In order to protect the identity of the patient, all archival clinical samples used in the study were coded and tested anonymously. The only available data were patient gender, age and immune status (if collected during the original study).

## Results and Discussion

HPV-125 was detected in 2004 in the biopsy specimen of a hand wart obtained from a 19 year-old immuno-competent male patient at the Department of Dermatovenereology, University Medical Centre, Ljubljana, Slovenia. It was initially identified as a putative novel HPV genotype by the use of HVP2/B5 primers [13], [14], designed to detect various cutaneous HPVs, with which a 474-bp sequence of the L1 gene was amplified and sequenced. This partial L1 sequence was deposited in GenBank as strain SIBX9 (Acc. No. AJ810860) in August 2004 [16]. In addition to HPV-125, the original clinical sample also contained genotypes HPV-8 and HPV-22, as identified by the use of Ma/Ha [27], and CPI/CPIIs [28] primers and sequencing, respectively.

By using PCR amplification, the complete genome of HPV-125 was cloned and deposited in the Reference Centre for Papillomaviruses in Heidelberg, Germany, where the genomic sequence of HPV-125 was independently confirmed and the novel genotype was assigned its name in October 2009, according to the consecutive number of characterization.

The full genome of HPV-125 was determined to be 7,809-bp in length with a GC content of 46.4%. Phylogenetic comparisons of the HPV-125 complete L1 sequence with L1 sequences of 116 HPV genotypes place HPV-125 within the *Alphapapillomavirus* species 2, closest to HPV-3 and HPV-28 (Figure 1). Additional pairwise alignment of HPV-125 L1 nucleic acid and amino acid sequences with corresponding sequences of HPV-3 and HPV-28 (Table 1) revealed that HPV-125 is most closely related to HPV-3.

**Table 1:** Percentage similarity between E6, E7, E1, E2, L1, L2 genes of HPV-125 and closely related HPVs from species 2 of *Alphapapillomaviruses*.

HPV-125	HPV-3	HPV-10	HPV-28	HPV-29	HPV-77	HPV-94	HPV-117
**E6**							
nt	84.8^a^	79.9^a^	83.2^a^	71.8^a^	76.7^a^	80.8^a^	77.9^a^
aa	82.4	73.8	83.0	65.1	74.5	77.9	72.5
**E7**							
nt	90.2^a^	86.4	90.9	81.0	81.0	89.4	86.4
aa	81.8	80.7	86.4	73,6	73.6	85.2	78.4
**E1**							
nt	88.6	81.0	86.0	80.9	80.1	80.6	80.9
aa	87.5	79.8	88.1	82.7	80.6	80.3	80.2
**E2**							
nt	86.4	77.8	84.3	75.0	75.7	78.7^a^	79.7
aa	82.3	74.0	79.2	69.2	70.0	73.5	74.5
**L1**							
nt	85.4	80.0	83.9	74.4^a^	74.3^a^	79.3	80.3
aa	90.8	86.4	88.7	81.9	80.0	84.0	85.4
**L2**							
nt	85.6	79.9	82.9	74.8	75.0^a^	75.0	76.7
aa	90.1	85.3	87.6	78.6	77.4	80.9	82.0

nt – nucleotide.

aa – aminoacid.

a ORFs not equal in length – similarity was calculated from the first common ATG of the two genotypes.

**Figure 2:**
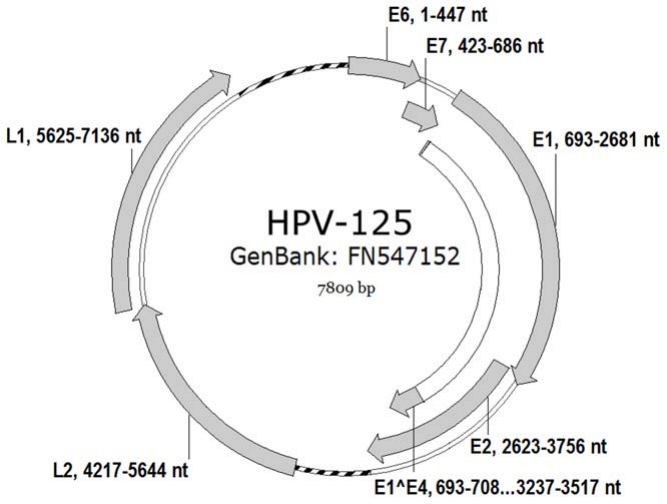
Genomic organisation of HPV-125. Genomic positions of viral genes E6, E7, E1, E2, E4 (E1∧E4), L1 and L2 are indicated next to the respective gene. The non-coding regions located between L1 and E6 (LCR); and E2 and L2 are indicated with a dotted line.

**Figure 3:**
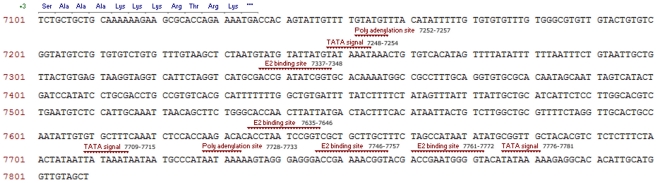
Characteristic features of the long control region (LCR) of HPV-125. The genomic locations of E2 binding sites (ACC-N_5–7_-GGT), polyadenilation sites (AATAAA), and TATA signals (TATAAA or TATA(A/T)A(A/T)) are indicated on the coding strand.

Further sequence analysis revealed that HPV-125 has a genomic organization typical of the *Alphapapillomavirus* genus (Figure 2) and contains genes coding for five early proteins E6, E7, E1, E2 and E4 and two late capsid proteins L1 and L2. The genome of the HPV-125 also contains two non-coding regions; the classic non-coding long control region (LCR) located between the L1 and E6 genes (nucleotide positions 7,137–7,809, length 673-bp) and a second non-coding region located between genes E2 and L2 (nucleotide positions 3,757–4,216, length 460-bp). Typical features of the LCR of HPV-125 are shown in Figure 3.

The percentage similarity of nucleotide and amino acid sequences of the six viral genes of HPV-125 shared by other HPV genotypes from *Alphapapillomavirus* species 2 is shown in Table 1. The nucleotide and amino acid sequences of the late genes L1 and L2 and the early gene E2 of HPV-125 showed the highest similarity to the corresponding genes of HPV-3. However, the early gene E7 of HPV-125 was more similar to E7 of HPV-28, than to E7 of HPV-3 (Table 1) in both nucleotide and amino acid sequences. The nucleotide sequence of the early gene E6 of HPV-125 showed the highest similarity to E6 of HPV-3, while its amino acid sequence was slightly more similar to the E6 protein of HPV-28. Similarly, the nucleotide sequence of the early gene E1 of HPV-125 showed the highest similarity to E1 of HPV-3, while the amino acid sequence was more similar to the E1 protein of HPV-28. Given that HPV-3, HPV-28 and HPV-125 are the most closely related genotypes within *Alphapapillomavirus* species 2, this could either reflect a recombination event or a different evolutionary divergence of the early and late genes of these three genotypes from their most recent common ancestor.

**Figure 4:**
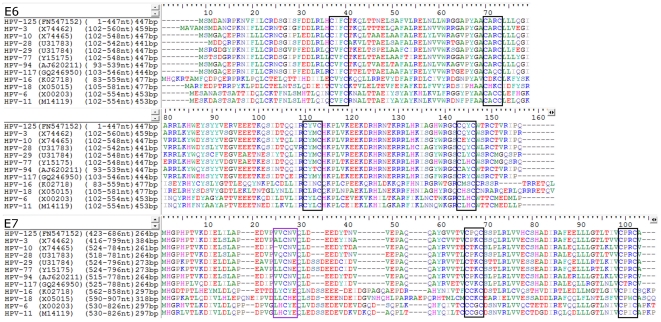
Amino acid alignment of HPV-125 E6 and E7 proteins with corresponding proteins of closely related genotypes from *Alphapapillomavirus* species 2 and genotypes HPV-16, HPV-18, HPV-6 and HPV-11. Black boxes indicate the location of zinc-binding domains (CxxC(x)_29_CxxC) in both E6 and E7 amino acid alignments. The purple box indicates the location of the pRb-binding core sequence (LxCxE), absent in several *Alphapapillomavirus* species 2 genotypes [30], including HPV-125.

*In silico* analysis showed that the E6 protein of HPV-125 contains two regular zinc-binding domains (CxxC(x)_29_CxxC) at amino acid positions 29 and 102, whereas the E7 protein of HPV-125 exhibits one such domain at position 50 (Figure 4). Like several other genotypes from *Alphapapillomavirus* species 2 [30], HPV-125 lacks the regular pRb-binding core sequence (LxCxE) [8], [10]–[12], [30], [31] within its E7 protein (Figure 4).

HPV-125, similarly to HPV-28 possesses an approximately 300-bp long E4 ORF at genomic location 3,215–3,517. However, it is likely that the E4 protein of HPV-125 is actually translated from a spliced mRNA consisting of the first few codons of the E1 ORF joined to the E4 ORF (E1∧E4 CDS consisting of genomic positions 693..708 and 3,237..3,517 nt) as was determined for other better-characterized *Alphapapillomaviruses*.

Unlike most HPV genotypes from genus *Alphapapillomavirus*, which have a mucosal tropism, members of *Alphapapillomavirus* species 2 (HPV-3, HPV-10, HPV-28, HPV-29, HPV-77, HPV-78, HPV-94 and HPV-117) exhibit cutaneous tropism [8]–[12], [14], [30]. The two genotypes of species 2, HPV-3 and HPV-10, were among the first HPVs to be characterized and were both isolated from scrapings of hand lesions, resembling flat warts, from immuno-compromised individuals with *epidermodysplasia verruciformis* [8]. In addition, these two genotypes were later found to be associated with flat warts of immuno-compromised, as well as immuno-competent individuals [9]. HPV-28 was first found in a case of butcher's warts [10], while HPV-29 was first detected in a common skin wart [11]. HPV-77 was first isolated from a benign wart but has also been found in several cases of dysplastic warts and SCC of the skin in renal-transplant recipients [12]. HPV-94 (DL40) was isolated from malignant skin tumor of a renal-transplant recipient [15]. HPV-117 was isolated from a common wart of an organ-transplant recipient, and was shown to be persistent over 24 months [30]. Based on the data acquired so far [8]–[12], [14], [30], members of *Alphapapillomavirus* species 2 seem to be associated predominantly with flat and common warts in immuno-competent individuals, while some genotypes belonging to this species also have the potential to cause malignant transformation in immuno-compromised hosts.

In order to assess the biological and clinical importance of HPV-125, a quantitative HPV-125 type-specific RT-PCR assay was developed, based on amplification of a 61-bp region of the HPV-125 L1 gene (Table S1). The standard curve of the assay showed an excellent correlation coefficient (R^2^ = 1.00) between the Ct (threshold) and the log of starting viral copy number, across at least seven orders of magnitude. The 95% limit of detection of the assay was 2.5 copies per reaction (range 1.7–5.7) and the intra and inter-assay coefficient of variation (CV (%) = (st.dev/mean Ct)*100) was 0.47 and 2.00 for 100 copies per reaction, and 1.15 and 2.15 for 10 copies per reaction, respectively. The assay showed no cross-reactivity with any of the tested non-targeted cutaneous HPV genotypes, including HPV-2, HPV-3, HPV-7, HPV-10, HPV-27, HPV-28, HPV-29, HPV-40, HPV-43, HPV-77, HPV-78 and HPV-91 in concentrations of 10^7^–10^8^ viral copies per reaction.

Because phylogenetic relationships between HPV genotypes do not necessarily always reflect their tissue tropism, and to assess the clinical significance of HPV-125, a representative collection of HPV-associated benign and malignant neoplasms and hair follicles were tested for the presence of HPV-125 using type-specific RT-PCR. The sample collection was chosen to represent various sites of HPV infection (mucosal/cutaneous) and possible outcomes of HPV infection (benign/malignant lesions)(Table 2). The panel of samples thus included the most important HPV-associated malignant neoplasms of mucosal (cervical squamous cell carcinoma) and cutaneous (SCC and BCC of skin) origin, the most important HPV-associated benign neoplasms of mucosal (genital warts, laryngeal papillomas) and cutaneous (common warts) origin and, in addition, hair follicles known to be a main reservoir of usually harmless ‘commensal’ HPV genotypes in immuno-competent individuals [27], [32].

**Table 2:** Clinical samples tested for the presence of HPV-125.

Tissue type	Sample type	No. of samples tested	No. (%) of HPV-125 positive samples
Mucosal			
	Genital warts	71	0 (0)
	Laryngeal papillomas	85	0 (0)
	Cervical cancer	104	0 (0)
Cutaneous			
	Common warts	102	2 (1.96)
	Squamous cell carcinoma of the skin	52	0 (0)
	Basal cell carcinoma of the skin	49	0 (0)
Hair follicles		138	1 (0.72)
Total		601	3 (0.50)

As shown in Table 2, none of the 260 mucosal samples tested (cervical carcinomas, genital warts and laryngeal papillomas) were positive for HPV-125. These findings indicate that HPV-125, in agreement with its phylogenetic placement within *Alphapapillomavirus* species 2, does not exhibit mucosal tropism.

Of the 102 samples of common warts tested, two samples (1.96%) contained HPV-125, indicating the cutaneous tropism of this genotype (Table 2). In the first sample, due to the presence of *Betapapillomavirus* genotypes HPV-8 and HPV-22, both known to be cutaneous HPV genotypes, the development of this common wart could not conclusively be attributed to HPV-125. In contrast, in the second sample, HPV-125 was the only HPV genotype detected, despite the use of several HPV genotyping assays, targeting a range of *Alphapapillomavirus* and *Betapapillomavirus* genotypes, which supports the causative role of HPV-125 in the development of this common wart (Table 3). In addition, in both HPV-125 positive common warts, a very high HPV-125 viral load was observed, indicating the presence of productive HPV-125 infection.

**Table 3:** Characteristics of HPV-125 positive samples and patients.

Sample type	Histology result	Age	Gender	Viral genomes/No. of human cells	Presence of other HPVs
Common wart	Verruca vulgaris	19	Male	2,61×10^3^/1	HPV-8, HPV-22
Common wart	Verruca vulgaris	24	Male	1,28×10^5^/1	None
Hair follicle	/	29	Male	Nd.	HPV-10, HPV-36

All HPV-125 positive samples were obtained from immuno-competent individuals.

Nd. – not determined.

HPV-125 was not detected in any of the 101 samples of malignant tumors of the skin tested. This finding may be due either to the lack of HPV-125 potential for malignant transformation, or to the rarity of this genotype in immuno-competent individuals. The results obtained indicate that HPV-125 does not seem to be an important non-melanoma skin cancer causing genotype, although the possibility of HPV-125 contributing to the development of malignant skin lesions in immuno-compromised individuals cannot be finally excluded, due to the rarity of the genotype and the number of samples tested so far.

Of the 138 hair follicle samples tested, a single hair follicle sample contained HPV-125. This sample also contained two additional HPV genotypes, HPV-10 and HPV-36, consistent with the current knowledge that HPVs are ubiquitous in hair follicles and that hair follicles represent a viral reservoir in immuno-competent individuals. Similarly to other members of *Alphapapillomavirus* species 2, it is possible that HPV-125 is significantly more frequent in immuno-compromised individuals [8]–[12], [14], [30]; however, this remains to be determined in further studies.

In conclusion, the characterization of the novel HPV genotype HPV-125 improves our knowledge about the cutaneotrophic *Alphapapillomavirus* species 2, which is phylogenetically nested deep within the predominantly mucosotrophic *Alphapapillomaviruses*. HPV-125 is a relatively rare HPV genotype with cutaneous tropism, ethiologically linked with sporadic cases of common warts.

## Supporting Information

Table S1 **Nt: nucleotide position, Cw: clockwise orientation, Ccw: counter-clockwise orientation.**
(DOC)
