# Supplementary material for: Characterization of a Novel Cutaneous Human Papillomavirus Genotype HPV-125
Source: PLoS One. 2011 Jul 21;6(7):e22414. doi: 10.1371/journal.pone.0022414 (PMC3141055; doi:10.1371/journal.pone.0022414)
Supplement: Table S1 — Nt: nucleotide position, Cw: clockwise orientation, Ccw: counter-clockwise orientation. (DOC) [file pone.0022414.s001.doc]

Table S1: Primers used for initial amplification of HPV-125 genomic DNA, primer-walking and preparation of reference clones.

| **Primer name** | **Sequence (5’- 3’)** | **Genomic location (nt)** | **Direction of primer** |
| --- | --- | --- | --- |
| 125-fpw1 | GTTAGAAGACACAGAAAATTCCACA | 5,999-6,023 | cw |
| 125-fpw2 | CGTGCCCTTAGACATTTGC | 6,278-6,296 | cw |
| 125-fpw3 | CAACCAACTGTTTGTAACTGTGG | 6,605-6,627 | cw |
| 125-fpw4 | CCAGTTTCCCCTTGGTAGG | 7,004-7,022 | cw |
| 125-fpw5 | GTATTGTGTGCTTTCAAATCTCC | 7,602-7,624 | cw |
| 125-fpw6 | GACGCCTGAAACACTGGG | 216-235 | cw |
| 125-fpw7 | GGTACAGAGGGGGACGAAT | 708-726 | cw |
| 125-fpw8 | GGGACGGACAGGAAGTGG | 877-894 | cw |
| 125-fpw9 | AAAGCATCCTGTGGAGTGTTAG | 1,296-1,317 | cw |
| 125-fpw10 | TTGTAGTGAAGTGTATGGGGAA | 1,715-1,736 | cw |
| 125-fpw11 | TTGGAGGATGAAAAATTGAAG | 2,442-2,462 | cw |
| 125-fpw12 | CTAGCGAACCGTTTAGATGTG | 2,632-2,652 | cw |
| 125-fpw13 | ATCTCGGGAAATGTGGGAC | 2,913-2,931 | cw |
| 125-fpw14 | GAAGGACCGGAGCAAAAG | 3,316-3,333 | cw |
| 125-fpw15 | GTGTTTGTTTTGGCTGTGTG | 3,821-3,840 | cw |
| 125-fpw16 | GCGTGCATCCGCCACA | 4,246-4,261 | cw |
| 125-fpw17 | CCTGCTTTTACAGAACCTTCC | 4,697-4,717 | cw |
| 125-fpw18 | CTACAAAGTATGGCAATGTCACC | 5,385-5,425 | cw |
| 125-fpw19 | AAGGTGTCCGCCTTTCAATA | 5,814-5,833 | cw |
| 125-rpw1 | ATACAAGGCGTCAGGGATTG | 6,430-6,449 | ccw |
| 125-rpw2 | CCATACCCAGTGTCCACCAT | 6,219-6,238 | ccw |
| 125-rpw3 | GGTAACCGCACCCTAAACAC | 5,838-5,857 | ccw |
| 125-rpw4 | AGGAGACACAAAGGGAATGG | 5,406-5,425 | ccw |
| 125-rpw5 | CTGTGAGGGGGAGTAAGGC | 5,020-5,038 | ccw |
| 125-rpw6 | AGACCTCCCAAATAAACACCA | 4,366-4,386 | ccw |
| 125-rpw7 | TGAGAACCAGGGGGTACATAG | 3,954-3,974 | ccw |
| 125-rpw8 | TTACATTAGTCAAAAATTCATCCC | 3,680-3,703 | ccw |
| 125-rpw9 | GGTCTCCATCATATCTAACTTCAA | 2,972-2,995 | ccw |
| 125-rpw10 | TGGTCCTCAAGATTGTTGCTAT | 2,687-2,708 | ccw |
| 125-rpw11 | GCATCGTCCAGCAAACC | 2,286-2,302 | ccw |
| 125-rpw12 | CATCCTTTACATACTTTGCCTG | 1,923-1,944 | ccw |
| 125-rpw13 | CCAAACACACACACCACCC | 1,438-1,456 | ccw |
| 125-rpw14 | CTTCCTGTCCGTCCCCTAC | 873-891 | ccw |
| 125-rpw15 | AAAGCTCCTCAAATGCCCT | 627-645 | ccw |
| 125-rpw16 | GTTTTCGGTCCCTCCCTAC | 7,736-7,754 | ccw |
| 125-rpw17 | ACATTCAGACGTGCCAAGG | 7,489-7,507 | ccw |
| 125-rpw18 | CCCACACCTACCTGCATCA | 7,030-7,048 | ccw |
| 125-rpw19 | CGTCATGTTAGTACTTCGTGTGG | 6,634-6,656 | ccw |
